# Functional brain network correlates of cardiorespiratory fitness and moderation by depression symptoms

**DOI:** 10.1007/s00429-025-02952-2

**Published:** 2025-06-05

**Authors:** Lauren R. Borrell, Andrew C. Venezia, J. Carson Smith

**Affiliations:** 1 Neuroscience and Cognitive Science Program, University of Maryland, College Park, MD 20742 USA; 2 Department of Health and Human Performance, The University of Scranton, Scranton, PA 18510 USA; 3 Department of Kinesiology, University of Maryland, College Park, MD 20742 USA

**Keywords:** Cardiorespiratory fitness, Functional connectivity, Depression, fMRI

## Abstract

Cardiorespiratory fitness may protect against or alleviate depression through neuroplastic mechanisms within disrupted brain networks underlying cognitive and affective symptoms. There is a scarcity of research examining the effects of cardiorespiratory fitness on functional brain networks in representative samples spanning adulthood. Further, little is known about the interaction of cardiorespiratory fitness with depression in these networks. To address this problem, this study made use of the NKI-Rockland sample dataset and magnetic resonance imaging to examine whether functional brain network features associated with cardiorespiratory fitness were moderated by depression symptom severity in a community sample of 217 participants (134 females, 83 males) between the ages of 18 and 71 (*M* = 43.8 years, *SD* = 16.2). Cardiorespiratory fitness was positively associated with whole-brain modularity, between-network connectivity of the central executive and salience networks, and within-network connectivity of the default mode, central executive, and salience networks. The association of cardiorespiratory fitness and default mode network connectivity was stronger in the presence of moderate-to-severe depression symptoms. Taken together, these observations lend support to prior work that has demonstrated the associations of cardiorespiratory fitness with reduced concurrent and prospective depression risk and symptom severity. Depression symptoms moderated default mode network connectivity, suggesting that cardiorespiratory fitness may differentially impact neural substrates for those with moderate-to-severe depression symptoms.

**Supplementary Information:**

The online version contains supplementary material available at 10.1007/s00429-025-02952-2.

## Introduction

Depressive disorders are the leading cause of disability worldwide (World Health Organization 2017). Considerable undertreatment magnifies the immense personal and economic burden of depression; even in high-income countries, only 1 in 5 individuals with a diagnosis of major depressive disorder receives minimally adequate treatment (Thornicroft et al. 2017). While pharmacotherapy may be effective for some, the majority of patients prescribed antidepressants are deemed treatment resistant within six months (Rush 2006). The use of adjunctive therapy with antipsychotics is costly, and confers the risk of numerous health-threatening side effects (Thase 2016). Taken together, these observations underscore the need to identify more cost-effective and sustainable treatment strategies for preventing and improving depression symptoms.

Physical activity, which constitutes voluntary movement that results in increased energy expenditure, is a promising nonpharmacological preventative and therapeutic treatment for depression (Heissel et al. 2023; Noetel et al. 2024; Rebar et al. 2015; Schuch et al. 2016a, b), but the mechanisms underlying the antidepressant effects of physical activity and exercise are largely unknown. A positive and measurable outcome of regular and consistent physical activity of sufficient intensity and volume is maintenance or improvement of cardiorespiratory fitness (CRF), which is an important physiological determinant of both physical and mental health, and a predictor of all cause mortality (Myers et al. 2024; Bouchard et al. 2015). Extant evidence strongly supports the conclusion that CRF is associated with fewer concurrent and incident depressive symptoms (Åberg et al. 2012; Baumeister et al. 2017; Papasavvas et al. 2016; Schuch et al. 2016a, b; Sui et al. 2009), making it a measurable target that can be used in the overall treatment or prevention of depression.

A triple network model has been proposed to explain the role of aberrant large-scale network topology and interconnectivity in psychopathology, including major depression (Menon 2011). This model involves the dysregulation of three core brain networks which underlie the complex cognitive and emotional process that are impacted in depression. These core brain networks are the default mode network (DMN), the frontoparietal central executive network (CEN), and the salience network (SAL) (Menon 2011). The DMN collectively refers to a set of brain regions that are reliably activated in a synchronous manner during periods of rest such as introspection and self-generated thought. Because of its role in introspective thought and rumination, the DMN has been of particular interest in the study of depression, as a hallmark of depression is perseveration on negative self-referential thoughts (Berman et al. 2011).

The frontoparietal CEN is responsible for numerous higher-order cognitive processes (e.g., working memory, attention, decision making) and is anchored in the dorsolateral prefrontal cortex (PFC) and lateral posterior parietal cortex (Seeley et al., 2007). A coordinate-based meta-analysis revealed hypoconnectivity within the CEN in depression (Kaiser et al. 2015). Alterations of FC between CEN and DMN have also been reported, though with inconsistent direction, likely reflecting impaired salience-related ‘switching’ and the disrupted coordination of CEN and DMN engagement (Kaiser et al. 2015; Menon 2011; Mulders et al., 2015). The brain’s SAL network is named for its critical role in detecting and integrating relevant sensory information, and has been linked to emotional control and switching between the DMN and CEN (Goulden et al., 2014; Seeley et al., 2007; Sridharan et al., 2008). Anchored in the dorsal ACC and operculo-insular cortex, the SAL shows increased activation during cognitive and affect processing, and is integral in orchestrating interactions between other core brain networks—the CEN and DMN, in particular (Menon and Uddin 2010). Hypo- and hyperconnectivity between distinct regions of the DMN and SAL have been reported in depression, and this dysregulation is proposed to underlie weak salience-related ‘switching’ and the altered inability to disengage ruminative and self-referential mental activity (Menon 2011). Within the triple network model proposed by Menon and colleagues (2011), the compromised ability of the SAL to appropriately assign saliency to external stimuli and internal mentalizations is a cornerstone of the dysregulated network interactions among DMN, CEN, and SAL. This dysregulation is reflected in aberrant coupling of these networks and imbalance resulting from failure to appropriately engage and disengage the internally-focused DMN and externally-focused CEN in relevant contexts.

Physical activity that is sufficient to maintain or improve CRF likely exerts positive effects on symptoms of depression through both psychosocial and biological mechanisms (Ross et al. 2023; Kandola et al. 2019). However, it has not been clearly established that higher levels of CRF protect against or alleviate depressive symptoms through neuroplastic mechanisms within brain networks implicated in the cognitive and affective pathology of the disorder. Much of the research in functional brain network correlates of CRF have been studied in the context of aging (Won et al. 2021). Few investigations have leveraged whole brain functional connectivity analyses, and graph theory applications, across adulthood or in studies of depressed individuals. The application of graph theory, a set of mathematical concepts developed to characterize complex networks, to study the brain has promoted the burgeoning field of network neuroscience. Graph theory is concerned with the understanding of complex systems, which can be described mathematically as graphs comprising individual components (‘nodes’) and the relations or connections between them (‘edges’). Zhang and colleagues (2011) applied graph theory techniques to ascertain differences in resting state network topology between drug-naïve first-episode major depressive disorder (MDD) patients and healthy controls. Comparisons of global small-world parameters, including clustering coefficient and path length, revealed significantly lower path length and increased global efficiency in MDD patients. These differences indicate a shift in network organization such that resting state networks in MDD more closely resemble random networks, hence disrupting the optimal balance between functional segregation and integration. Pairwise comparison of network connectivity, utilizing the network-based statistic for control of family-wise error rate, revealed a subnetwork comprising numerous DMN regions that was more intrinsically connected in MDD patients than controls. Later work by Zheng and colleagues (2015) investigated aberrant FC of large-scale brain networks within the triple network model framework. Intrinsic (i.e., within-network) and internetwork (i.e., between-network) FC of the DMN, CEN, and SAL were examined in a heterogenous group of MDD patients compared to non-depressed controls. In line with predictions of the triple network model, increased connectivity between the DMN and CEN was observed in conjunction with increased degree (i.e., high number of connections) of the right anterior insula, a critical hub of SAL. This hyperconnectivity of the anterior insula—a key orchestrator of salience mapping and orientation between the CEN and DMN—in MDD reflects its role as a potential focal point in disrupted modularity and dynamic functional segregation between networks (Menon 2011; Menon and Uddin 2010). Using graph theory, Ye and colleagues (2015) compared medication naïve MDD patients to healthy controls and found whole-brain network modularity — a measure of clustering into well-defined communities within a network — was significantly and positively correlated with depressive symptom scores. Various measures reflecting nodal centrality, which reflect the importance of individual nodes on facilitating information transfer between functionally segregated networks and generally indicate a node’s “hubness” in the context of a community of nodes or an entire network, revealed altered centrality in various brain regions in MDD, with the majority of nodes exhibiting modified centrality within the DMN and CEN.

Relatively few studies have examined the influence of CRF on network measures reflecting the organization of intrinsic brain networks. Much of the existing work is focused on CRF in the context of aging-related network dysregulation (Won et al. 2021). For example, a study by Voss and colleagues (2016) compared network characteristics associated with aging and CRF. The network-based statistic, a technique to identify effects within connected components of nodes and edges (Zalesky et al. 2010), revealed stronger connectivity within functionally coherent networks of younger adults. Conversely, between-network connections were stronger in older adults. These findings suggest dysregulation of the balance between functional segregation and integration of distinct networks in the older adult brain. Among older adults, Voss et al. (2016) reported positive associations between CRF and within-network functional connectivity, primarily in the DMN. CRF was negatively associated with the strength of several between-network connections, further suggesting that higher CRF may combat deleterious shifts in brain organization associated with aging. Although the Voss et al. (2016) study did not investigate the relationship between CRF and FC in individuals with depression, the study lends strong support that CRF is associated with FC in those at risk for deleterious shifts in brain organization.

Investigations linking CRF to functional brain network organization in young or middle-aged adults are scarce. However, one recent study utilized a modern multivariate method and graph theory to investigate individual differences in FC in healthy young adults (Talukdar et al. 2018) and reported CRF was associated with the multivariate functional connectivity profiles of eight widespread brain regions within prefrontal, middle temporal, and parietal lobes, cerebellum, and thalamus. Further analysis of connectivity profiles within atlas-defined networks revealed substantial involvement of CRF- associated regions across multiple intrinsic functional networks, with the most influence on core networks including the CEN and DMN (Talukdar et al. 2018). Linking brain to behavior, the strength of FC for CRF-sensitive brain regions within the CEN significantly predicted fluid intelligence scores. This cross-sectional study demonstrates in young adults that differences in functional brain network topology associated with CRF can be detected at an individual level. The widespread differences associated with CRF in this large and comprehensive study reflect the potential of higher CRF to impact and potentially protect multiple brain networks, which may have clinical implications for the prevention and or treatment of depression.

Existing applications of graph theoretical techniques to the functional connectome suggest a promising role of CRF in the organization of brain networks underlying specialized cognitive and affective processes. The relationship between CRF and FC in large-scale networks has yet to be investigated in a number of populations, including clinical populations and at-risk populations who may exhibit aberrant whole-brain topology. Research is needed to determine if the associations between CRF and large-scale network connectivity are modified by depressive symptoms in these populations. Using a publicly available large-scale data set (Nathan Kline Institute Rockland Sample; https://www.nki.rfmh.org/study/rockland-sample/), the purpose of this study was to determine the functional brain network correlates of CRF and the extent to which these associations are moderated by depression symptoms. This study was part of a dissertation project completed by L.R.B (Weiss 2020; abstract available at 10.13016/mhvb-jlft). We examined modularity based on prior research suggesting modularity is altered in depression and may be related to depression symptoms (Ye et al. 2015). We hypothesized that CRF would be associated with higher modularity of the whole-brain network. Based on the triple network model (Menon 2011), we specifically examined the association of CRF with functional connectivity within and between the DMN, CEN, and SAL and whether these associations were moderated by depression symptoms. Drawing from existing work that has examined associations of these constructs with functional brain network connectivity, we expected that CRF would be associated with graph theoretical metrics. Given the relatively few studies that have examined these metrics, particularly in samples including young and middle-aged adults, we did not have directional hypotheses about the effects of CRF or depression symptoms on within-network and between-network functional connectivity.

## Methods

The publicly available enhanced Nathan Kline Institute Rockland Sample (NKI-RS) is an ongoing effort to create a large-scale community sample of participants across the lifespan (http://fcon_1000.projects.nitrc.org/indi/enhanced; Nooner et al. 2012). The NKI-RS is comprised of several studies that share a core research protocol; therefore, several phenotypic and neuroimaging measures are collected from participants enrolled in any one or more of these studies. Anonymized neuroimaging and phenotypic (e.g., behavioral, physical) data from this core research protocol for approximately 1,500 participants between the ages of 6–85 were available at the time of data extraction. The full phenotypic data release was accessed under an authorized NKI-RS Data Usage Agreement [PI: J Carson Smith] and extracted from the Longitudinal Online Research and Imaging System (LORIS) Database. Further, use of these data was approved by the University of Maryland College Park Institutional Review Board.

### Subsample selection and demographics

The behavioral variables of interest for these analyses included estimated V̇O_2max_ and BDI-II score. V̇O_2max_ was estimated using a modified Åstrand-Ryhming submaximal cycle ergometer test (see Supplemental Materials; Astrand & Ryhming, 1954). The presence and severity of depressive symptoms was assessed with the Beck Depression Inventory-II (BDI-II; Beck et al. 1996). The BDI-II is a self-report questionnaire and comprises 21 items designed to assess depressive symptoms including but not limited to sadness, feelings of failure and worthlessness, anhedonia, self-criticism, agitation, and changes in energy or appetite. For each item, participants select the statement that best describes their feelings during the past two weeks on a four-point scale ranging from 0 to 3. Score on the BDI-II is calculated from the sum of these items. Possible scores are between 0 and 63, with 0–13 considered minimal, 14–19 considered mild, and 20–63 considered moderate-to-severe depression (Beck et al. 1996).

Complete details of participant inclusion and exclusion in the current sample can be found in the Supplemental Materials. Briefly, of those participants included in the NKI database aged between 18 and 85 years old during data collection, a subset of *n* = 790 participants was identified with complete diagnostic summary, medical, and drug screening data. Because planned analyses required complete data for each case entered into multiple linear regression models, participants from this initial sample were included only if PA measures, V̇O_2max_, BDI-II score, and both structural and functional neuroimaging data were available (*n* = 372). Participants were also excluded if > 28 days elapsed between collection of behavioral or neuroimaging data (*n* = 97). Quality control of the raw and preprocessed functional volumes resulted in exclusion of 49 additional participants prior to conducting group analyses. An additional nine participants were excluded based on casewise regression diagnostics. Details of these procedures are described in the subsequent sections. The final sample included *n* = 217 participants (134 females, 83 males) between the ages of 18 and 71 (*M* = 43.8, *SD* = 16.2; 76% white, 13% black or African-American, 9% Asian, 2% other race; 8% Hispanic; see Figure S2 in Supplemental Materials).

Within this subsample, *n* = 41 participants had a positive diagnostic history of depressive disorder or mood disorder exhibiting depressive symptoms including current or remitted MDD, dysthymic disorder, adjustment disorder with depressed mood, or depressive disorder not otherwise specified. Fourteen participants self-reported current and regular use of one (*n* = 12) or more (*n* = 2) psychotropic medications for a duration ≥ 3 months. These medications included serotonin-specific reuptake inhibitors (SSRIs; *n* = 6), serotonin-norepinephrine reuptake inhibitors (SNRIs; *n* = 3), norepinephrine-dopamine reuptake inhibitors (NDRIs; *n* = 1), benzodiazepines (*n* = 4), stimulants (*n* = 3), tricyclic antidepressants (*n* = 1), and sedative-hypnotics (*n* = 1). Depression symptom severity assessed on the BDI-II was higher for participants with a positive diagnostic history (*M* = 12.0, *SD* = 9.5) than those with a negative diagnostic history (*M* = 4.6, *SD* = 4.5; *t*(43.983) = 4.844, *p* <.001).

### Functional MRI data acquisition and preprocessing

Blood oxygen level-dependent functional images were acquired in the axial plane using a T2*-weighted multiband echoplanar imaging pulse sequence with contiguous interleaved slices (TE = 30ms, TR = 1400ms, TR delay = 0ms, flip angle = 65°, field-of-view = 224 × 224 mm, matrix = 112 × 112, number of slices = 64, voxel size = 2.0mm^3^, phase encoding A > > P, GRAPPA accel. factor = 4, number of volumes = 404, acquisition time = 8:06). This sequence was developed for the Washington University-University of Minnesota consortium of the Human Connectome Project, and provided to NKI-RS for state-of-the-art acquisition of whole-brain functional MRI (Xu et al. 2012).

### Functional connectivity graph analyses

All fMRI data were run through standard quality control and preprocessing pipelines in preparation for whole brain functional connectivity analysis (see Supplemental Materials).

#### Functional brain parcellation scheme

Functional brain network nodes were defined using a widely-used brain atlas (Power et al. 2011). This atlas was derived using a combination of meta-analytic and functional connectivity mapping techniques and comprises 264 regions of interest widely distributed across cortical, subcortical, and cerebellar brain regions. These regions of interest are modeled as spherical nodes representing individual elements of large-scale brain organization. These nodes comprise a number of brain networks including but not limited to the DMN, CEN, and SAL. Prior work has applied this atlas to examine interactions among core networks of interest in the present project (Chen et al. 2016; Lydon-Staley et al. 2019). For the current project, a modified version of the original 264-node graph was created. This modified version excluded a subnetwork of 28 nodes which were assigned ad hoc based on uncertain community membership during atlas creation (Power et al. 2011). For analyses focused on specific brain networks, the DMN and SAL were defined in accordance with labels assigned in Power et al. (2011), while the CEN comprised the network identified by the authors as the *fronto-parietal task control network*.

Subject-specific network node masks were created for the extraction of residual BOLD signal from the preprocessed functional volumes. Spherical regions of interest (10 mm in diameter) were created in MNI space centered on coordinates provided by Power et al. (2011) and excluding the 28-node subnetwork described above. Each participant’s standard-to-native-space warp was calculated by concatenating the previously calculated affine alignment with the affine and nonlinear spatial transformation warp. The standard-space region of interest volumes were warped to each subject’s standard space by applying the inverse transformation of this concatenation.

#### Construction of functional connectivity networks

The average residual BOLD signal within each region of interest (i.e., network node) was extracted for calculation of network edges. Edge weights of subject-specific networks were defined as the Fisher’s r-to-z transformed Pearson product-moment cross-correlations of the average BOLD time series calculated between network nodes. A 236 × 236 undirected, weighted, and signed correlation matrix was constructed such that *A*_*ij*_ was defined as the r-to-z transformed cross-correlation between nodes *i* and *j*.

### Functional brain network metrics

#### Modularity

**Fig. 1:**
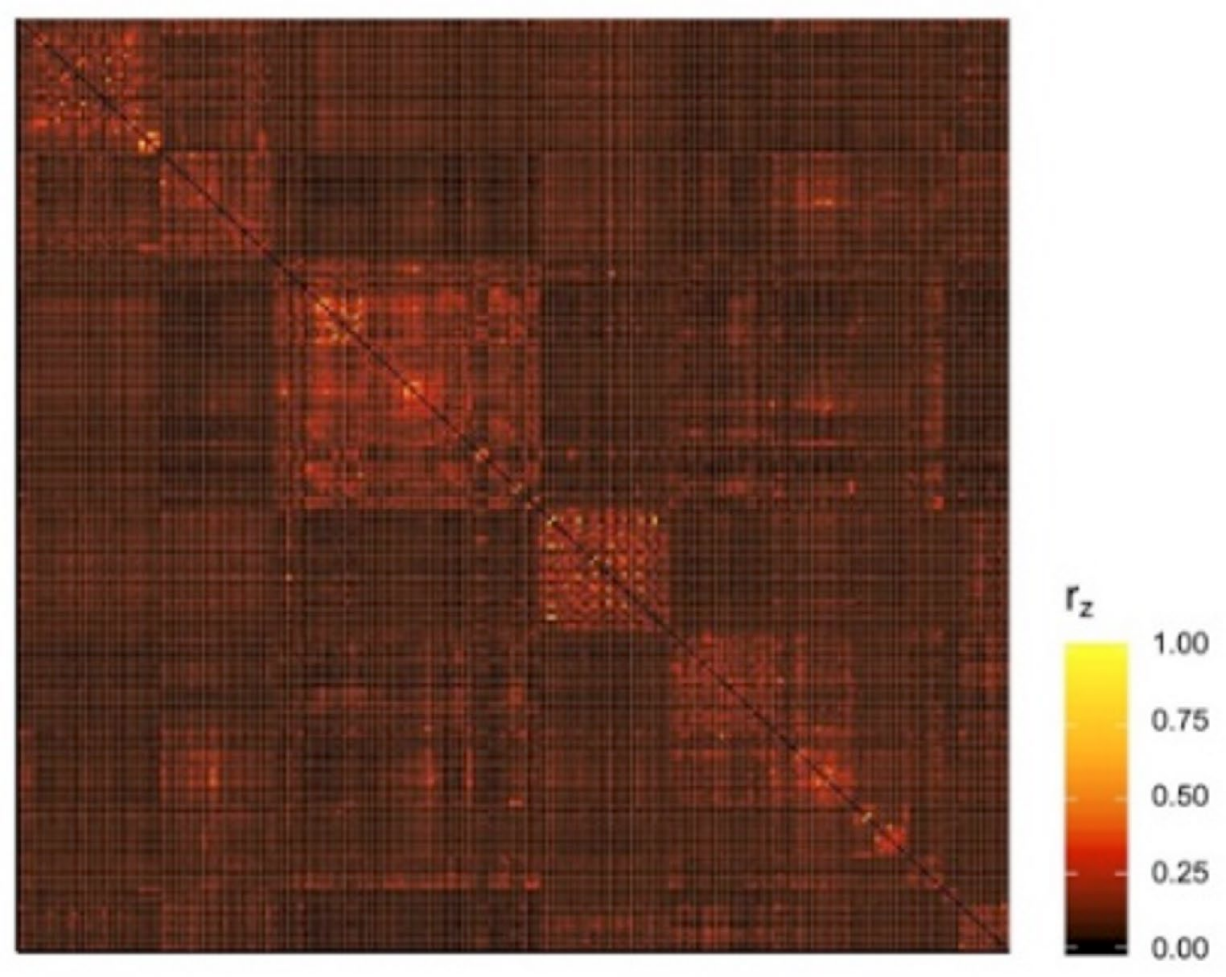
Group-averaged adjacency matrix representing functional connectivity of whole-brain network defined using Power (2011) atlas. Negative weights were removed (i.e., set to 0) for modularity analysis

Modularity was calculated from each participant’s whole-network adjacency matrix. For the calculation of modularity, community membership was assigned based on atlas-defined clusters of networks derived from independent samples (Power et al. 2011). This method served to ensure consistent community membership of network nodes across participants. Negative edges were removed from adjacency matrices for this calculation, thus creating an undirected, unsigned, and weighted matrix for each participant. The group-averaged adjacency matrix is presented in Fig. 1. Calculation of modularity based on community assignment was conducted within the *igraph* package for R (https://igraph.org/r/), within which the modularity index *Q* is calculated as:

$$\:Q=\:\frac{1}{2m}\:\times\:\:\sum\:_{ij}\left({A}_{ij}-\:\frac{{k}_{i}{k}_{j}}{2m}\right)\:\delta\:\:\left({c}_{i},\:{c}_{j}\right)$$

Here, $\:m$ is the number of edges within the adjacency matrix, $\:{A}_{ij}$ is the edge weight of vertex $\:ij$, and $\:{k}_{i}{k}_{j}/2m$ represents the weight of edges linking nodes in the same community by chance (Clauset et al. 2004). The function $\:\delta\:\:\left({c}_{i},\:{c}_{j}\right)$ is equal to one if two nodes belong to the same module (i.e., community) and zero otherwise. This equation is therefore quantifying the difference between intramodule connectivity in a given network compared to that expected by chance.

#### Within-network and between-network functional connectivity

**Fig. 2:**
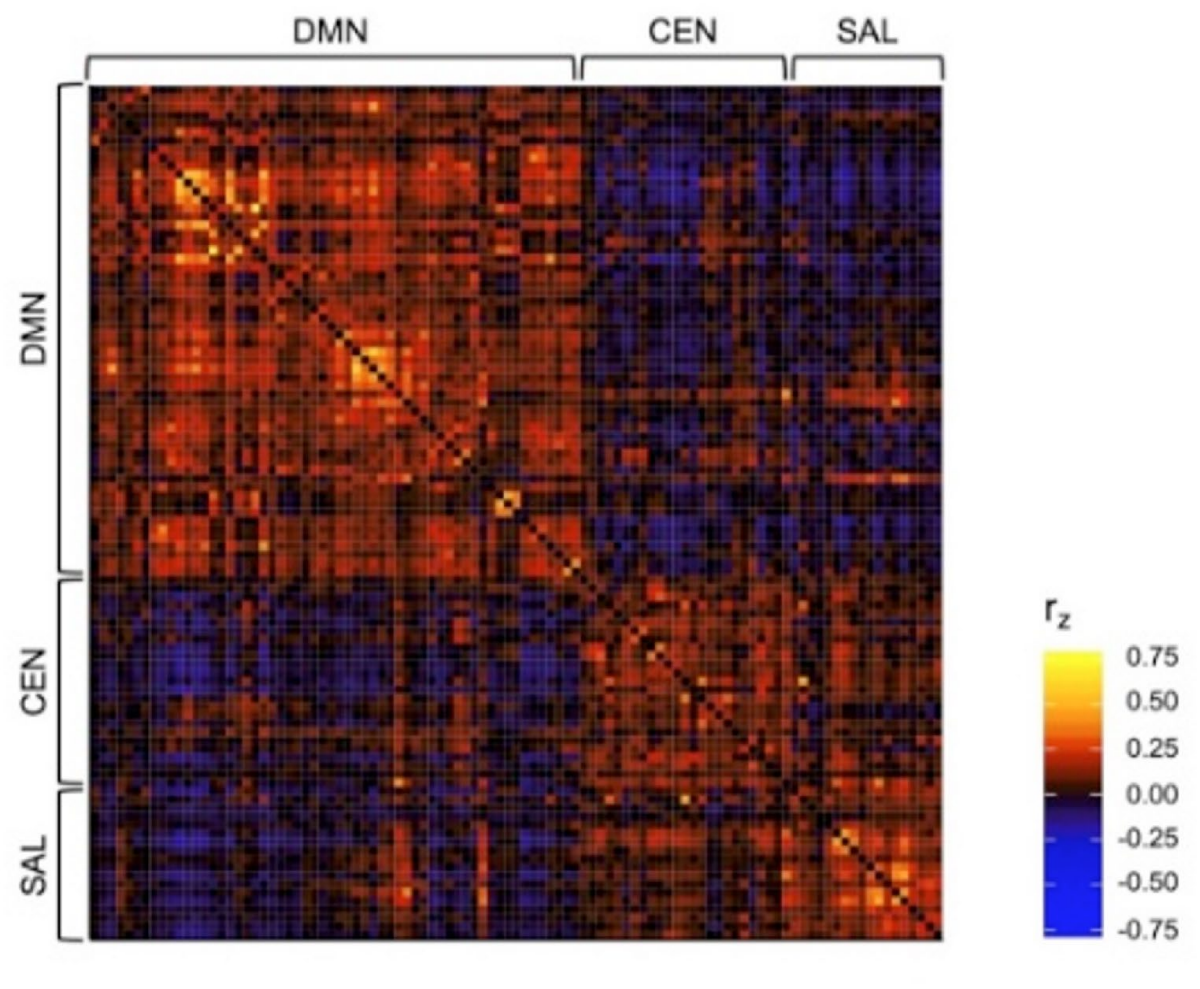
Group-averaged adjacency matrix representing functional connectivity of DMN, CEN, and SAL defined using Power (2011) atlas

To assess associations CRF with functional brain network topology in the context of the triple-network model, average functional connectivity was calculated within and between the DMN, CEN, and SAL. Negative edges were retained in adjacency matrices for this calculation, thus creating an undirected, signed, and weighted matrix for each participant. While it is common practice to threshold correlation matrices in order to retain only a fraction of the strongest connections, adjacency matrices were not thresholded for these calculations due to the potential neurobiological relevance of weak and negatively correlated (i.e., anticorrelated) functional connectivity for examining whole-brain network topology (Chen et al. 2016; Sporns and Betzel 2016). The group-averaged adjacency matrix representing the DMN, CEN, and SAL is presented in Fig. 2. Within-network functional connectivity was calculated for each participant as the average of all edges (nij) within the DMN (DMN_w_), CEN (CEN_w_), and SAL (SAL_w_) for each participant. Between-network functional connectivity was calculated as the average of all edges between nodes of the DMN and nodes of the CEN (DMN-CEN_b_), between nodes of the CEN and nodes of the SAL (CEN-SAL_b_), and between nodes of the SAL and nodes of the DMN (SAL-DMN_b_).

### Multiple linear regression analyses

Association of V̇O_2max_ with functional brain network metrics and potential interactions with BDI-II score were examined separately using a hierarchical linear regression approach. Sex was entered as a dummy variable with males as the reference group (i.e., male = 0, female = 1). First-step models included predictors for V̇O_2max_, BDI-II score, age, age^2^, and sex. Second-step models incorporated the interaction term (i.e., V̇O_2max_ × BDI-II) to determine the extent to which depression symptom severity moderated the association of V̇O_2max_ on the metric of interest. All regression analyses, including diagnostic review and exclusion of *n* = 9 participants based on case-wise evaluation, were conducted in accordance with an a priori statistical analysis plan.

#### Regression diagnostics and assumptions

Casewise diagnostics were conducted to identify potentially influential data points in the first-step regression models. Centered leverage values were examined within index plots using the heuristic value 2(*p* + 1)/*n*, where *p* is the number of predictors, as a guideline for identifying points with large leverage. Studentized and studentized deleted residuals and Bonferroni-corrected *t* tests of studentized deleted residuals were examined to identify problematic outliers. For evaluation of influence, particular attention was paid to Cook’s *D* and DFFITS (i.e., global influence statistics) using the heuristic Cooks *D* > 0.5 and DFFITS > 2/n as guidelines for identifying points with large influence on the multivariate regression. DFBETAS (i.e., a local influence statistic) was also examined using the heuristic DFBETAS > 2/n with emphasis on points demonstrating large influence on the regression coefficients for V̇O_2max_. Note that these heuristic values were not applied as strict cut-offs. Comprehensive review of these diagnostic tests and visualizations guided the identification of nine cases with problematic influence within multiple regression models. Based in in-depth evaluation, cases were removed prior to re-estimation of regression models to mitigate the likely impact on regression parameters. Casewise diagnostics were reviewed after removal of these cases and indicated satisfactory resolution of problems with leverage and influence.

The regression assumptions of linearity, normality, homoscedasticity, independence, and were evaluated across all first- and second-step models using a combination of diagnostic tests and data visualization. The linearity assumption was visually assessed by plotting each predictor and predicted values against residuals in combination with Tukey’s test of nonadditivity (Tukey 1949). These methods confirmed the assumption of linearity was reasonable (all *p >*.05). The normality assumption was visually assessed using Q-Q plots of the unstandardized residual. This review confirmed that the conditional residuals of regression models were normally distributed. The assumption of homoscedasticity was evaluated by plotting model residuals against fitted values. This procedure confirmed that the assumption of homoscedasticity was reasonably met. The Durbin-Watson test for correlated residuals was conducted to evaluate the assumption of independence. These tests confirmed that the residuals were not autocorrelated (all *p* >.05), and therefore that the assumption of independence was reasonably met. Multicollinearity diagnostics including variance inflation factors and tolerance values did not detect issues of multicollinearity in any of the reported models.

#### Power analyses

Power analyses were conducted in G*Power (v.3.1; (Faul et al. 2007) to determine the sample size necessary to detect effects of interest in multiple linear regression models. The first analysis was conducted to determine the required sample size to reject the omnibus null hypothesis (*R*^2^ ≠ 0) in first-step linear regression models with the following parameters: *f*^2^ = 0.1, *ɑ* = 0.05, power (1-*β*) = 0.8, number of predictors = 5. This protocol yielded a required sample size of *n* = 134. Examining total sample size as a function of *f*^2^ suggested the ability to detect effects as small as approximately *f*^2^ = 0.05 with *n* = 217. The second analysis was conducted to determine the required sample size to detect the effect of a single regression coefficient in first-step linear regression models with the following parameters: *f*^2^ = 0.1, *ɑ* = 0.05, power (1-*β*) = 0.8. This protocol yielded a required sample size of *n* = 81.

The third analysis was conducted to determine the required sample size to detect the effect of the interaction term in second-step regression models (*R*^2^) with the following parameters: *f*^2^ = 0.1, *ɑ* = 0.05, power (1-*β*) = 0.8, number of tested predictors = 1, total number of predictors = 6. This protocol yielded a required sample size of *n* = 81. Examining total sample size as a function of *f*^2^ suggested the ability to detect effects as small as approximately *f*^2^ = 0.035 with *n* = 217. These analyses confirmed the appropriateness of conducting the proposed analyses with *n* = 217 participants comprising a sufficient sample size to reject null hypotheses of interest.

## Results

Descriptive statistics and distributions of measured variables are presented in Table 1. Multiple linear regression models examining the associations and interactions of V̇O_2max_ and BDI-II on functional brain network metrics are presented in Table 2.

**Table 1:** Descriptive statistics of measured variables for subsample included in functional brain network analyses

	Males	Females	All Participants
	*n* = 83	*n* = 134	*n* = 217
Variable	*M* ± *SD*	*M* ± *SD*	*M* ± *SD*
Age (Years)	40.6 ± 17.4	45.8 ± 15.4	43.8 ± 16.3
BDI-II Score	6.16 ± 6.46	5.5 ± 5.3	5.8 ± 5.8
Estimated V̇O_2max_(mL⋅kg^−1^⋅min^−1^)	42.78 ± 11.08	28.49 ± 9.21	33.96 ± 12.13
Total PA (MET⋅min⋅wk^− 1^)	4620 ± 5066	4659 ± 4258	4644 ± 4572

**Table 2:** Moderated multiple regression models testing the effects and interaction of V̇O_2max_ and BDI-II on functional brain network metrics

DMN_w_	Partial Model					Moderation Model				
	b	SE b	𝛽	t	*p* value	b	SE b	𝛽	t	*p* value
V̇O_2max_ (mL⋅kg^−1^⋅min^−1^)	1.24E-03	2.70E-04	0.392	4.595	**< 0.001**	7.50E-04	3.30E-04	0.236	2.270	**0.024**
BDI-II Score	4.55E-04	4.27E-04	0.068	1.066	0.288	-2.54E-03	1.25E-03	-0.378	-2.021	**0.045**
Age (Years)	-7.94E-04	9.65E-04	-0.336	-0.823	0.412	-7.06E-04	9.53E-04	-0.299	-0.740	0.460
Age^2^ (Years^2^)	1.35E-05	1.10E-05	0.500	1.231	0.220	1.26E-05	1.09E-05	0.464	1.157	0.249
Sex	3.73E-02	6.20E-03	0.472	6.010	**< 0.001**	3.73E-02	6.12E-03	0.471	6.084	**< 0.001**
V̇O_2max_ x BDI-II						8.37E-05	3.31E-05	0.495	2.530	**0.012**
*R* ^2^	0.159					*R* ^2^	0.184			
Adj. *R*^2^	0.139					Adj. *R*^2^	0.160			
*F*(∆*R*^2^)	7.969	**< 0.001**				*F*(∆*R*^2^)	6.402	**0.012**		
**CEN**_**w**_										
V̇O_2max_ (mL⋅kg^−1^⋅min^−1^)	8.19E-04	3.00E-04	0.243	2.732	**0.007**	6.62E-04	3.72E-04	0.197	1.782	0.076
BDI-II Score	-6.73E-04	4.73E-04	-0.095	-1.423	0.156	-1.63E-03	1.41E-03	-0.229	-1.154	0.250
Age (Years)	1.55E-03	1.07E-03	0.619	1.449	0.149	1.58E-03	1.07E-03	0.630	1.472	0.142
Age^2^ (Years^2^)	-1.97E-05	1.22E-05	-0.684	-1.614	0.108	-2.00E-05	1.22E-05	-0.695	-1.636	0.103
Sex	1.86E-02	6.88E-03	0.222	2.703	**0.007**	1.86E-02	6.89E-03	0.222	2.699	**0.008**
V̇O_2max_ x BDI-II						2.67E-05	3.72E-05	0.149	0.719	0.473
*R* ^2^	0.082					*R* ^2^	0.084			
Adj. *R*^2^	0.060					Adj. *R*^2^	0.058			
*F*(∆*R*^2^)	3.771	**0.003**				*F*(∆*R*^2^)	0.516	0.473		
**SAL**_**w**_										
V̇O_2max_ (mL⋅kg^−1^⋅min^−1^)	1.33E-03	3.85E-04	0.308	3.444	**0.001**	1.40E-03	4.78E-04	0.326	2.934	**0.004**
BDI-II Score	4.53E-04	6.08E-04	0.050	0.746	0.457	9.08E-04	1.81E-03	0.100	0.501	0.617
Age (Years)	-5.89E-04	1.37E-03	-0.184	-0.429	0.669	-6.02E-04	1.38E-03	-0.188	-0.437	0.663
Age^2^ (Years^2^)	6.06E-06	1.57E-05	0.165	0.387	0.699	6.21E-06	1.57E-05	0.169	0.396	0.693
Sex	1.59E-02	8.83E-03	0.149	1.802	0.073	1.59E-02	8.85E-03	0.149	1.798	0.074
V̇O_2max_ x BDI-II						-1.28E-05	4.78E-05	-0.056	-0.267	0.790
*R* ^2^	0.071					*R* ^2^	0.072			
Adj. *R*^2^	0.049					Adj. *R*^2^	0.045			
*F*(∆*R*^2^)	3.241	**0.008**				*F*(∆*R*^2^)	0.071	**0.790**		
**DMN-CEN**_**b**_										
V̇O_2max_ (mL⋅kg^−1^⋅min^−1^)	2.33E-04	2.23E-04	0.095	1.044	0.298	1.34E-04	2.76E-04	0.055	0.485	0.628
BDI-II Score	4.32E-04	3.52E-04	0.083	1.228	0.221	-1.67E-04	1.05E-03	-0.032	-0.159	0.874
Age (Years)	-2.97E-04	7.96E-04	-0.162	-0.373	0.710	-2.79E-04	7.97E-04	-0.153	-0.350	0.727
Age^2^ (Years^2^)	5.12E-06	9.07E-06	0.244	0.565	0.573	4.93E-06	9.09E-06	0.235	0.543	0.588
Sex	1.42E-02	5.11E-03	0.233	2.784	**0.006**	1.42E-02	5.12E-03	0.232	2.779	**0.006**
V̇O_2max_ x BDI-II						1.68E-05	2.77E-05	0.128	0.606	0.545
*R* ^2^	0.047					*R* ^2^	0.049			
Adj. *R*^2^	0.024					Adj. *R*^2^	0.021			
*F*(∆*R*^2^)	2.076	0.070				*F*(∆*R*^2^)	0.368	0.545		
**CEN-SAL**_**b**_										
V̇O_2max_ (mL⋅kg^−1^⋅min^−1^)	6.72E-04	2.23E-04	0.272	3.016	**0.003**	7.62E-04	2.76E-04	0.309	2.756	**0.006**
BDI-II Score	-3.74E-04	3.52E-04	-0.072	-1.062	0.290	1.68E-04	1.05E-03	0.032	0.160	0.873
Age (Years)	-7.80E-05	7.95E-04	-0.043	-0.098	0.922	-9.40E-05	7.97E-04	-0.051	-0.118	0.906
Age^2^ (Years^2^)	4.42E-06	9.06E-06	0.210	0.488	0.626	4.60E-06	9.08E-06	0.218	0.506	0.613
Sex	9.90E-03	5.11E-03	0.161	1.937	0.054	9.90E-03	5.12E-03	0.161	1.934	0.054
V̇O_2max_ x BDI-II						-1.52E-05	2.77E-05	-0.115	-0.548	0.584
*R* ^2^	0.055					*R* ^2^	0.057			
Adj. *R*^2^	0.033					Adj. *R*^2^	0.030			
*F*(∆*R*^2^)	2.463	**0.034**				*F*(∆*R*^2^)	0.301	0.584		
**SAL-DMN**_**b**_										
V̇O_2max_ (mL⋅kg^−1^⋅min^−1^)	-1.24E-04	2.68E-04	-0.043	-0.465	0.642	-2.31E-04	3.32E-04	-0.080	-0.697	0.487
BDI-II Score	1.20E-04	4.22E-04	0.020	0.284	0.777	-5.28E-04	1.26E-03	-0.086	-0.419	0.675
Age (Years)	-2.73E-04	9.54E-04	-0.127	-0.286	0.775	-2.54E-04	9.56E-04	-0.118	-0.266	0.791
Age^2^ (Years^2^)	5.46E-06	1.09E-05	0.221	0.502	0.616	5.25E-06	1.09E-05	0.212	0.482	0.630
Sex	-3.21E-03	6.13E-03	-0.044	-0.524	0.601	-3.21E-03	6.14E-03	-0.045	-0.523	0.601
V̇O_2max_ x BDI-II						1.81E-05	3.32E-05	0.117	0.546	0.585
*R* ^2^	0.014					*R* ^2^	0.015			
Adj. *R*^2^	-0.010					Adj. *R*^2^	-0.013			
*F*(∆*R*^2^)	0.595	0.704				*F*(∆*R*^2^)	0.299	0.585		
**Modularity (** ***Q*** **)**										
V̇O_2max_ (mL⋅kg^−1^⋅min^−1^)	4.62E-04	1.28E-04	0.320	3.614	**< 0.001**	4.20E-04	1.59E-04	0.291	2.649	**0.009**
BDI-II Score	-1.74E-04	2.02E-04	-0.057	-0.860	0.391	-4.30E-04	6.02E-04	-0.141	-0.713	0.476
Age (Years)	-2.71E-04	4.56E-04	-0.252	-0.594	0.553	-2.64E-04	4.58E-04	-0.245	-0.576	0.565
Age^2^ (Years^2^)	3.11E-06	5.20E-06	0.252	0.599	0.550	3.03E-06	5.21E-06	0.246	0.581	0.562
Sex	1.13E-02	2.93E-03	0.315	3.867	**< 0.001**	1.13E-02	2.94E-03	0.315	3.860	**< 0.001**
V̇O_2max_ x BDI-II						7.17E-06	1.59E-05	0.093	0.452	0.652
*R* ^2^	0.093					*R* ^2^	0.094			
Adj. *R*^2^	0.072					Adj. *R*^2^	0.068			
*F*(∆*R*^2^)	4.339	**0.001**				*F*(∆*R*^2^)	0.204	0.652		

### V̇O_2max_ and BDI-II on functional brain network metrics

First-step (i.e., partial) models included predictors for V̇O_2max_, BDI-II score, age, age^2^, and sex. Second-step (i.e., moderation) models incorporated the interaction term (i.e., V̇O_2max_ × BDI-II) to determine the extent to which depression symptom severity moderated the association of V̇O_2max_ on the functional brain network metric of interest.

#### Modularity

The partial model explained 9.3% of the variance in modularity (*F*(5, 211) = 4.339, *p* =.001). Both V̇O_2max_ (𝛽 = 0.320, *t*(210) = 3.614, *p* <.001) and sex (𝛽 = 0.315, *t*(210) = 3.867, *p* <.001) were significant predictors of modularity. Holding all else constant, a 1-SD increase of V̇O_2max_ was associated with a 0.320-SD increase of modularity. Addition of the V̇O_2max_ × BDI-II interaction term did not explain a significantly larger portion variance in modularity (∆*R*^2^ = 0.001, *F(1*, 229) = 0.422, *p* =.517). Total PA (MET⋅min⋅wk^−1^) was not associated with any between network measures.

#### Within-network connectivity

**Fig. 3:**
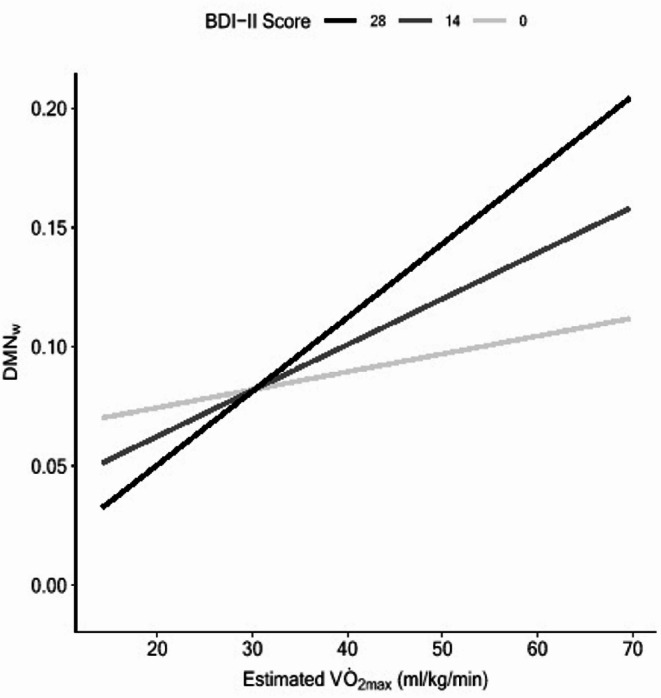
Interaction plot depicting the regression line of DMN_w_ on V̇O_2max_ at discrete levels of BDI-II score

Within the DMN, the partial model explained 15.9% of the variance in DMN_w_ (*F*(5, 211) = 7.969, *p* <.001). Both V̇O_2max_ (𝛽 = 0.392, *t*(210) = 4.595, *p* <.001) and sex (𝛽 = 0.472, *t*(210) = 6.010, *p* <.001) were significant predictors of DMN_w_. Addition of the V̇O_2max_ x BDI-II interaction term explained a significantly larger portion of variance in DMN_w_ (∆*R*^2^ = 0.025, *F*(1, 229) = 6.402, *p* =.012). This moderation is illustrated in Fig. 3. The Johnson-Neyman test indicated that the simple slope between V̇O_2max_ and DMN_w_ was significant across all values of BDI-II score. Simple slopes were calculated for BDI-II scores of 0, 14, and 28. The unstandardized simple slope for participants with a BDI-II score of 0 was 0.0007 (*SE* = 0.0003, 95%CI [0.0001, 0.0014], *p* =.024). The unstandardized simple slope at a BDI-II score of 14 was 0.0019 (*SE* = 0.0004, 95%CI [0.0012, 0.0027], *p* <.001) and at a BDI-II score of 28 was 0.0031 (*SE* = 0.0008, 95%CI [0.0016, 0.0046], *p* <.001). Evaluation of confidence intervals indicates that the relationship between V̇O_2max_ and DMN_w_ is significantly more positive at higher levels of depression symptom severity (i.e., BDI-II of 28) relative to low or absent depression symptoms (i.e., BDI-II of 0).

Within the CEN, the partial model explained 8.2% of the variance in CEN_w_ (*F*(5, 211) = 3.771, *p* =.003). Both V̇O_2max_ (𝛽 = 0.243, *t*(210) = 2.732, *p* =.007) and sex (𝛽 = 0.222, *t*(210) = 2.703, *p* =.007) were significant predictors of CEN_w_. Holding all else constant, a 1-SD increase of V̇O_2max_ was associated with a 0.243-SD increase of CEN_w_. Addition of the V̇O_2max_ × BDI-II interaction term did not explain a significantly larger portion variance in CEN_w_ (∆*R*^2^ = 0.001, *F(1*, 229) = 0.422, *p* =.517). Total PA (MET⋅min⋅wk^− 1^) was negatively associated with only CEN_w_ (𝛽 = -0.160, *t*(210) = -2.384, *p* =.018), but not any of the other within-network measures.

Within the SAL, the partial model explained 7.1% of the variance in SAL_w_ (*F*(5, 211) = 3.241, *p* =.008). V̇O_2max_ (𝛽 = 0.308, *t*(210) = 3.444, *p* =.001) was a significant predictor of SAL_w_. Holding all else constant, a 1-SD increase of V̇O_2max_ was associated with a 0.308-SD increase of SAL_w_. Addition of the V̇O_2max_ × BDI-II interaction term did not explain a significantly larger portion variance in SAL_w_ (∆*R*^2^ = 0.001, *F(1*, 229) = 0.071, *p* =.790).

#### Between-network connectivity

Between the CEN and SAL, the partial model explained 5.5% of the variance in CEN-SAL_b_ (*F*(5, 211) = 2.463, *p* =.034). V̇O_2max_ (𝛽 = 0.272, *t*(210) = 3.106, *p* =.003) was a significant predictor of CEN-SAL_b_. Holding all else constant, a 1-SD increase of V̇O_2max_ was associated with a 0.272-SD increase of CEN-SAL_b_. Addition of the V̇O_2max_ x BDI-II interaction term did not explain a significantly larger portion variance in SAL_w_ (∆*R*^2^ = 0.002, *F(1*, 229) = 0.301, *p* =.584).

Between the DMN and CEN, neither the partial model (*R*^2^ = 0.047, *F*(5, 211) = 2.076, *p* =.070) nor the moderation model (*R*^2^ = 0.049, *F*(6, 210) = 1.786, *p* =.103) explained a significant portion of the variance in DMN-CEN_b_. Between the SAL and DMN, neither the partial model (*R*^2^ = 0.014, *F*(5, 211) = 0.595, *p* =.074) nor the moderation model (*R*^2^ = 0.015, *F*(6, 210) = 0.544, *p* =.774) explained a significant portion of the variance in DMN-SAL_b_. Total PA (MET⋅min⋅wk^-1^) was not associated with any between network measure.

## Discussion

Depression has been consistently associated with dysregulation of large-scale functional brain networks, and emerging work demonstrates associations of CRF with graph theoretical measures of functional brain network organization (Won et al. 2021). We examined functional brain network correlates of CRF in a representative sample spanning adulthood. We hypothesized that CRF would be associated with higher modularity of the whole-brain network but did not make directed hypotheses about within- and between-network connectivity of the DMN, CEN, and SAL due to the limited number of studies that have examined these measures in comparable samples. Drawing from existing work that has examined associations of these constructs with functional brain network connectivity, we expected that CRF would be associated with these graph theoretical metrics. We further investigated whether associations of CRF with whole-brain modularity or within- and between-network FC of core large-scale networks was moderated by depression symptoms.

We observed a pattern of results across modularity and core network metrics such that V̇O_2max_ and sex emerged as consistent predictors of these outcomes. Beyond the variance accounted for by age, sex, and depression symptoms, V̇O_2max_ was positively associated with whole-brain modularity and within-network connectivity of the DMN, CEN, and SAL. Further, depression symptoms moderated the association of CRF with DMN_w_ such that the strength of this association increased in conjunction with BDI-II score. Between-network connectivity of the CEN and SAL was positively associated with V̇O_2max_, but no further associations with between-network metrics were observed.

Our modularity results are in line with our predictions as we expected that CRF would be positively associated with modularity. Here, V̇O_2max_ was positively predictive of whole-brain functional network modularity beyond the influence of age, sex, and BDI-II score. Modularity represents the clustering of distinct subnetworks within a larger network. Within human brain networks, modularity is critical and serves to segregate networks and thereby promote specialized brain functions (Wig 2017). Loss of modularity is therefore associated with imbalanced segregation and integration dynamics of functional brain networks, as can be observed even in healthy aging (Chan et al. 2014).

Modularity has recently been proposed as a predictor of behavioral intervention-related plasticity (Gallen and D’Esposito 2019). One recent study found that baseline modularity was predictive of gains in executive function across multiple arms of a 6-month exercise intervention, though did not directly examine associations of CRF and modularity (Baniqued et al. 2018). Multiple studies have demonstrated disrupted segregation and abnormal recruitment of ‘out-of-community’ nodes as a hallmark of brain network dysfunction in depression (Berman et al. 2011; Gong and He 2015; Jiang et al. 2019; Lord et al. 2012). These observations are interesting to consider in the context of our CRF-related findings, as it may suggest that interventions aimed at increasing CRF improve the preparation of functionally specialized brain networks to reconfigure in response to behavioral intervention. Modularity may therefore be a key functional measure to examine in the context of exercise aimed to improve CRF as an adjunctive therapy to manage or alleviate depression symptoms (Kerling et al. 2015; Mura et al. 2014).

Since modularity is informed in part by within-community connections of various subnetworks, the positive association of V̇O_2max_ with metrics representing within-network FC of the DMN, CEN, and SAL aligns with these results. Within all three core networks of interest, within-network FC was positively associated with V̇O_2max_ beyond variance accounted for by age, sex, and BDI-II score. We also detected moderation within the DMN such that the positive association of CRF with DMN_w_ was stronger as depression symptoms increased. The association of CRF with DMN_w_ is consistent with prior cross-sectional studies (Voss et al. 2010a, b, 2016) and with randomized controlled trials suggesting that exercise training interventions can increase DMN FC in older adults (Chirles et al. 2017; Voss, 2010a, b). It is not clear why individuals with higher DMN_w_ still reported being severely depressed. One interpretation of the higher positive association of CRF with DMN_w_ in those who report higher depression is that low CRF in these individuals is exceptionally deleterious to within-network connectivity in the DMN. To our knowledge, this is the first study to observe interacting effects of CRF and depression symptoms on FC within the DMN, a brain network exhibiting dysfunction in depression (Brakowski et al. 2017; Menon 2011).

The associations of V̇O_2max_ with CEN_w_ and SAL_w_ connectivity are also consistent with work demonstrating that CRF accounts for inter-individual variance within these networks in healthy adults (Talukdar et al. 2018). Recent meta-analytic evidence cites hypoconnectivity within the CEN as a characterizing functional brain network feature in MDD, though this effect does not appear to be moderated by depression symptom severity (Kaiser et al. 2015). We also observed positive associations of CRF and CEN-SAL_b_ FC. Critically, disrupted information flow between the SAL and CEN is related to compromised cognitive function in the context of the triple network model (Menon and Uddin 2010). While the current study did not examine associations of CRF-related between-network FC with behavior, future work should examine these relationships paired with behavioral measures of executive function in the context of depression (Snyder 2013). While we did find one negative association between total PA and CEN_w_, caution is warranted in its interpretation given that PA was not associated with any of the other outcomes, including modularity. We have previously reported that CRF, but not PA, is a predictor of brain structural integrity (transitivity, which is analogous to modularity) in a different sample of older adults (Callow and Smith 2023), which is consistent with the current findings.

There are limitations which warrant caution when interpreting results of this study. Of course, the current study is cross-sectional in nature and aimed to explore the association of CRF with FC of core brain networks in a representative sample spanning adulthood. While we further examined the moderating influence of depression symptoms on functional brain network activity, randomized controlled trials are necessary to draw causal conclusions about the effects of CRF on these outcomes and the impact of depression. Based on core networks identified in the triple network model, average FC measures were calculated to reflect information flow within and between networks underlying depression pathology. While we identified a number of associations between these measures and CRF, we did not observe an association of depression symptoms with these outcomes in any of the tested models. This may be in part due to the relatively low occurrence of depression symptoms in this sample. While this study includes participants with and without a history or diagnosis of depression, self-reported symptoms were positively skewed and generally in the minimal to mild range. Moreover, we operationalized depression throughout this project as depression symptom severity assessed using the Beck Depression Inventory-II. While those participants with a positive diagnostic history of depressive disorder did exhibit higher concurrent depression symptoms, this measure does not adequately capture the impact of depression over time. Further, depression is a heterogenous disorder comprising a number of symptoms and symptom profiles across participants; this heterogeneity is not accounted for by use of a total symptom severity score. Participants also varied on current medication status and duration of use, as we did not exclude for stable psychotropic use. While this decision was made to ultimately include a greater number of participants with a wide range of depression symptoms, medication history and status may impact brain functional outcomes. Further, while our network measures reflect functional correlations within and between core brain networks that were defined using volume-based normalization from the Power 2011 atlas, we lack the resolution to draw conclusions about specific nodes within networks of interest, which could be improved in future studies by using surface-based normilzation procedures (Brodoehl et al. 2020). Differences in modularity and network topology may be driven by changes in a small number of nodes influencing information flow within a network; therefore, further investigation into node-specific associations with PA and CRF, in particular, is necessary to identify the source(s) of altered network organization.

Extensive observational and experimental research demonstrates the potential of CRF to protect against or alleviate depression symptoms, but the underlying neural substrates of these effects are not well understood. Further, little is known about how these associations vary as a function of depression symptom severity. In this cross-sectional study, we demonstrate that CRF is associated with brain network modularity, within-network connectivity, and between-network connectivity of core functional networks implicated in the pathology of depression. These associations were observed in a representative community sample spanning adulthood, and were not explained by differences in age, sex, or concurrent depression symptoms. In the DMN, a network supporting introspective and self-directed thought, the association of CRF with network connectivity was stronger for those with more severe depressive symptoms. Taken together, these findings suggest that CRF may promote functional brain network modularity and strengthen within-network connectivity. Because our cross-sectional design precludes making any causal conclusions about fitness-related changes of functional brain network activity and organization, future experimental work should examine the association of exercise-related improvements in CRF with functional brain network and behavioral outcomes in depressed and non-depressed samples.

## Conclusions

It has been assumed, though never conclusively demonstrated, that physical activity (PA) and improvements in CRF may protect against or alleviate the depression syndrome through neuroplastic mechanisms acting on disrupted brain networks underlying its cognitive and affective symptoms. Indeed, there is a scarcity of research examining the influence of CRF on structural and functional brain networks in large samples across the lifespan. Further, little is known about the interaction of CRF with depression symptoms on these networks. An understanding of these interactions at a single point in time is an important first step in predicting potential longitudinal associations between these factors as mediators of the well documented therapeutic effects of exercise interventions on clinical depression. We examined the association of CRF with functional brain network modularity and measures of network connectivity focused on core subnetworks implicated in the triple network model. We observed an association of CRF with brain network modularity and within-network connectivity of the default mode, central executive, and salience networks. In the default mode network, a network supporting introspective and self-directed thought, the association of CRF with network connectivity was stronger for those with more severe depressive symptoms, potentially suggesting that low CRF is even more detrimental to FC connectivity in this network with higher symptoms of depression. Taken together, these observations lend support to prior work that has demonstrated the associations of CRF with reduced concurrent and prospective depression risk and symptom severity. Depression symptoms moderated default mode network connectivity, suggesting that CRF may differentially impact neural substrates for those with moderate-to-severe depression symptoms.

## Electronic supplementary material

Below is the link to the electronic supplementary material.

Supplementary Material 1

## Data Availability

These data were publicly available from the NKI Rockland sample: https://www.nki.rfmh.org/study/rockland-sample/.
