# Supplementary material for: Functional brain network correlates of cardiorespiratory fitness and moderation by depression symptoms
Source: Brain Struct Funct. 2025 Jun 5;230(6):85. doi: 10.1007/s00429-025-02952-2 (PMC12141363; doi:10.1007/s00429-025-02952-2)
Supplement: Supplementary file 1 — Supplementary Material 1 [file 429_2025_2952_MOESM1_ESM.docx]

Supplementary Information

“Functional Brain Network Correlates of Cardiorespiratory Fitness and Moderation by Depression Symptoms”

Lauren R. Borrell^1^, Andrew C. Venezia^2^, and J. Carson Smith^1,3,†^

^1^ University of Maryland, Neuroscience and Cognitive Science Program, College Park, MD, 20742, USA

^2^ The University of Scranton, Department of Health and Human Performance, Scranton, PA, 18510, USA

^3^ University of Maryland, Department of Kinesiology, College Park, MD, 20742, USA

^†^ Corresponding author: J. Carson Smith, Ph.D., University of Maryland, Department of Kinesiology, College Park, MD, 20742, USA. Email: [carson@umd.edu](mailto:carson@umd.edu)

# Enhanced Nathan Kline Institute Rockland Sample

The publicly available enhanced Nathan Kline Institute Rockland Sample (NKI-RS) is an ongoing effort to create a large-scale community sample of participants across the lifespan (http://fcon_1000.projects.nitrc.org/indi/enhanced; Nooner et al., 2012). Relevant data for the current dissertation included advanced neuroimaging, physiological assessments (e.g., cardiovascular fitness testing), and psychometric assessments. The NKI-RS is comprised of several studies that share a core research protocol; therefore, a number of phenotypic and neuroimaging measures are collected from participants enrolled in any one or more of these studies. Anonymized neuroimaging and phenotypic (e.g., behavioral, physical) data from this core research protocol are currently available for approximately 1,500 participants between the ages of 6-85. Use of these data for this dissertation project was approved through the exempt review process by the University of Maryland College Park Institutional Review Board.

Phenotypic data from 1,087 adults (18-85 years old) were available and extracted from LORIS. While the majority of these participants completed only one core research protocol (i.e., baseline) visit, a small subset of participants completed a second (n = 91) or third (n = 1) baseline visit after some delay. For these participants, the most recent baseline visit was selected for subsequent cleaning and transformation. The following sections include expanded detail of measurements and assessments used for this dissertation project. Data collection methods and scoring procedures are as reported by NKI-RS except where additional or modified calculations are noted.

# Phenotypic Data Extraction and Transformation

The full phenotypic data release was accessed under an authorized NKI-RS Data Usage Agreement [PI: J Carson Smith] and extracted from the Longitudinal Online Research and Imaging System (LORIS) Database. This data release includes diagnostic, psychiatric, behavioral, and laboratory assessments. Information was collected from participants using various published or study-specific questionnaires and instruments. Data points that were of interest in the current study as primary variables of interest, descriptive variables, or criteria for inclusion are described in the sections below. This project was approved under exempt review by the Institutional Review Board at the University of Maryland College Park.

## Demographic Information

Demographic and Handedness Information. Participants self-reported demographic information including age, sex, race, and ethnicity. With the exception of age and sex, demographic information was gathered only for descriptive purposes. Age was used as a criterion for inclusion, and both age and sex were included as control variables and covariates across multiple aims of this project.

Educational Attainment. Socioeconomic status was assessed using the Hollingshead Four-Factor Index of Socioeconomic Status (Hollingshead, 1975). This questionnaire was administered by an interviewer, and includes items regarding educational attainment, occupational prestige, marital status, and employment status. The participant’s highest grade completed is coded into an education index representing a standardized value for educational attainment (1 = less than 7^th^ grade, 2 = up to 9^th^ grade, 3 = partial high school up to 10^th^ or 11^th^ grade, 4 = high school graduate, 5 = some college and/or ≥ 1 year of specialized training, 6 = standard college or university degree, 7 = graduate or professional training). For the current project, this educational index was gathered to be included as a control variable in the structural equation model.

## Diagnostic and Medical Information

Diagnostic Summary. Qualified study staff completed a diagnostic summary for each participant following administration and review of the Structured Clinical Interview for DSM-IV-TR Axis I Disorders – Non-Patient Edition (First, Spitzer, Gibbon, & Williams, 2002) and Adult ADHD Clinical Diagnostic Scale (Adler & Cohen, 2004). These semi-structured interviews were administered to all adult participants in the NKI-RS to assess current and past episodes of psychopathology and attention-deficit hyperactivity disorder. Summary information comprised diagnoses with categorical qualifiers referencing status of diagnosis (e.g., past, current, recurrent). Diagnostic summary information was used to describe the study sample, specifically the prevalence of clinical depression, and to enforce exclusion criteria based on the presence of a(ny) relevant diagnosis.

Medical History and Medications. Information regarding current or past medical and psychiatric conditions was collected using multiple instruments designed by NKI-RS study staff. The medical history questionnaire is a self-report form designed to construct a comprehensive record of each participant’s medical and psychiatric history. Participants respond *Yes* or *No* to a list of possible medical issues with some items requesting further elaboration. Additionally, study staff directly questioned participants regarding current illnesses or health problems and recorded all responses. Self-reported medical and psychiatric conditions were combined with diagnostic summary information to enforce inclusion and exclusion criteria.

Participants were directly questioned about current medications by study staff. If participants reported routine use of any medication(s) or supplement(s), the name and duration of medication use was recorded. All medications and supplements reported were manually reviewed. Participants were not excluded from the current study for stable use of medications; however, medications with known effects on heart rate (e.g., beta-blockers) were coded for integration into the cardiovascular fitness testing data cleaning procedures. Briefly, medications endorsed by participants were cross-referenced within DrugBank (http://www.drugbank.ca/) for relevant information on drug target and mechanism of action. Chronic-use medications with direct action on beta- or alpha-adrenoceptors and with high or intermediate selectivity for cardiac muscle or otherwise documented impact on heart rate were coded as exclusionary (Appendix, Suppl. Table 1). These determinations were made in consultation with published guidelines for exercise testing (American College of Sports Medicine, 2018). Current and stable use of antidepressants, benzodiazepines, antianxiety, or other psychotropic medications was coded for descriptive purposes and for exclusion where duration of medication use was < 3 months.

Blood and Urine Lab Testing. Blood and urine samples were analyzed by the Office of Mental Health Clinical Laboratories at the Nathan S. Kline Institute for Psychiatric Research. Outcomes include routine panels and screenings for benzodiazepines, tricyclic antidepressants, cocaine, methamphetamines, methadone, oxycodone, phencyclidine, barbiturates, tetrahydrocannabinol, ecstasy, and amphetamines. These data were coded both for descriptive purposes and for assessment of exclusion criteria (e.g., positive illicit drug screen).

## Physical Activity

Intensity-specific and total physical activity (PA) were assessed using the International Physical Activity Questionnaire (IPAQ; Craig et al., 2003). The IPAQ was developed by an International Consensus Group on Physical Activity Measurement to obtain estimates of PA level across countries and cultures with acceptable validity (Booth, 2000). The NKI-RS uses a self-administered long version of the IPAQ that prompts participants to report on PA in the past week. This version of the IPAQ comprises a set of 27 items probing individuals about multiple domains of PA including work-related PA, transportation-related PA, household and gardening activities, and activity in leisure time for sport, exercise, and recreation. Within each domain, participants report on time spent engaging in low-intensity PA (i.e., walking), moderate-intensity PA, and vigorous-intensity PA. Illustrative examples of activities within these intensity-defined categories are included within relevant items of the questionnaire. A continuous measure of energy expenditure is computed by multiplying time spent in each category of activity by its energy requirements in metabolic equivalents (METs). The resulting estimate of total weekly energy expenditure (MET⋅min⋅wk^-1^) is a convenient and standardized measure of PA volume (American College of Sports Medicine, 2018).

This long form of the IPAQ exhibits excellent test-retest reliability for measurement of total PA in adults (Craig et al., 2003; Silsbury, Goldsmith, & Rushton, 2015). Measurement of PA on the IPAQ has demonstrated moderate concurrent validity against accelerometry (*ρ* = 0.3-0.55) and energy expenditure estimates from the gold standard doubly labeled water method (r = 0.33; Boon, Hamlin, Steel, & Ross, 2010; Craig et al., 2003; Hagströmer, Oja, & Sjöström, 2007; Maddison et al., 2007). The measurement properties of this IPAQ form are comparable or preferable to other available self-report measures (Silsbury et al., 2015).

Guidelines for scoring IPAQ data are provided by the IPAQ Research Committee (IPAQ Research Committee, 2005). Briefly, weekly duration at each intensity (i.e., low, moderate, vigorous) and within each domain (i.e., work, transportation, leisure time, etc.) was calculated as the product of number of days reported and time spent each day. PA was operationalized as both intensity-specific and total MET⋅min⋅wk^-1^ calculated from responses on the IPAQ. To calculate an intensity-specific PA score (MET⋅min⋅wk^-1^), the sum of values within each intensity category is multiplied by MET values corresponding to walking (3.3 METs), moderate PA (4.0 METs), and vigorous PA (8.0 METs). Total PA scores were obtained by calculating the sum of values across walking, moderate-intensity, and vigorous-intensity items.

In accordance with published scoring guidelines, cases with missing data on any item(s) contributing to the total PA score were excluded for analyses utilizing total PA. Similarly, intensity-specific PA scores were excluded for participants with missing data on any item(s) contributing to a given intensity-specific PA score. Therefore, it is possible that participants had intensity-specific PA scores available for light-intensity, moderate-intensity, or vigorous-intensity PA; however, total PA was only available for participants with complete data within all of these intensity categories. Also pursuant to the data cleaning guidelines, data were excluded for participants reporting an average of more than 960 minutes (16 hours) of physical activity per day. While the scoring guidelines specify rules for truncation of low-, moderate-, or vigorous-intensity timing values exceeding 180 minutes per day, data were not truncated for this project to avoid creating censored—rather than continuous—variables.

## Cardiorespiratory Fitness

Submaximal Cycle Ergometer Test. CRF was operationalized by measuring heart rate (HR) during a modified Åstrand-Ryhming submaximal cycle ergometer test (Astrand & Ryhming, 1954; Legge & Banister, 1986; Siconolfi, Cullinane, Carleton, & Thompson, 1982) and subsequently extrapolating estimated maximal oxygen consumption rate (V̇O_2max_). While direct measurement of V̇O_2max_ is the gold standard for assessment of CRF, submaximal exercise testing reflects CRF at a lower cost and with reduced risk of adverse events (American College of Sports Medicine, 2018). Participants were instructed to cycle for 6 min at 70 revolutions per minute on a recumbent cycle ergometer (RBK 815, Precor, Woodinville, WA) set to an age- and sex-determined resistance level (Appendix, Suppl. Table 2). HR was measured prior to and throughout testing with a pulse oximeter applied to the finger. Cycling was monitored to ensure participants maintained a speed of 68-72 revolutions per minute. Final HR (HR_final_) and work rate (WR_final_; watts) were recorded at the end of the 6-minute interval. If the participant was unable to complete the full 6-minute interval, failure time and final HR at failure were recorded.

Estimates of V̇O_2max_ were extrapolated based on HR_final_ and WR_final_ using equations derived from the modified Åstrand nomogram (Appendix, Cullinane, 1988; Siconolfi et al., 1982). This method of calculating estimated V̇O_2max_ shows high correspondence between measured V̇O_2max_ for both men (*r* = 0.88) and women (*r* = 0.87) across multiple age groups (Siconolfi et al., 1982). Calculated values of estimated V̇O_2max_ were corrected for age (Cullinane, 1988). In order to make meaningful comparisons among participants, this age-corrected estimate was converted to and expressed relative to body weight in units of mL⋅kg^-1^⋅min^-1^.

Cardiorespiratory fitness data were excluded where participants failed to complete the full 6-minute interval or reported taking HR-altering medications (Appendix, Suppl. Table 1). Values falling outside the range of 12.0-90.0 mL⋅kg^-1^⋅min^-1^ were excluded based on published reference values on V̇O_2max_ for men and women across relevant age groups (American College of Sports Medicine, 2018; Kaminsky, Arena, & Myers, 2015; Kaminsky, Imboden, Arena, & Myers, 2017). In a subset of n = 171 participants with both measures available, Pearson’s correlations were conducted to examine the agreement of estimated V̇O_2max_ derived from the submaximal cycle ergometer test with V̇O_2max_ measured during a maximal cycling protocol with respiratory measures. This analysis demonstrated reasonable agreement of the submaximal V̇O_2max_ estimate with the metabolically-assessed measure, with the submaximal estimation generally overestimating CRF (*r*(169) = .639, 95%CI [0.54, 0.721], *p* < .001).

### Calculation of Estimated V̇O_2max_

Estimates of V̇O_2max_ were calculated based on HR_final_ and WR_final_ using the following sets of equations derived from the modified Åstrand nomogram (Cullinane, 1988):

WR_final_ (kgm⋅min^-1^) = WR_final_ (watts) × 6.12

Men:

%V̇O_2max_ = 0.769 × HR_final_ − 48.5

V̇O_2max_ (L⋅min^-1^) = [ (0.00212 × WR_final_ (kgm⋅min^-1^) + 0.299) / %V̇O_2max_ ] × 100

Women:

%V̇O_2max_ = 0.769 × HR_final_ − 56.1

V̇O_2max_ (L⋅min^-1^) = [ (0.00193 × WR_final_ (kgm⋅min^-1^) + 0.326) / %V̇O_2max_ ] × 100

Absolute values of estimated V̇O_2max_ were further corrected for age (Cullinane, 1988):

Men:

V̇O_2max.corr_ (L⋅min^-1^) = 0.348 × V̇O_2max_ (L⋅min^-1^) − 0.035 × age (yr) + 3.011

Women:

V̇O_2max.corr_ (L⋅min^-1^) = 0.302 × V̇O_2max_ (L⋅min^-1^) − 0.019 × age (yr) + 1.593

In order to make meaningful comparisons among participants, this estimate was converted to and expressed relative to body weight:

V̇O_2max_ (mL⋅kg^-1^⋅min^-1^) = V̇O_2max.corr_ (L⋅min^-1^) × 1000 / mass (kg)

Depression Symptom Presence and Severity

The presence and severity of depressive symptoms was assessed with the Beck Depression Inventory-II (BDI-II; Beck, Steer, & Brown, 1996). The BDI-II is a self-report questionnaire designed to measure the presence and severity of symptoms, and one of the most widely used self-report measures of depressive symptoms. The BDI‐II was developed to correspond to DSM‐IV criteria for diagnosing depressive disorders and comprises 21 items designed to assess depressive symptoms including but not limited to sadness, feelings of failure and worthlessness, anhedonia, self-criticism, agitation, and changes in energy or appetite. For each item, participants select the statement that best describes their feelings during the past two weeks on a four-point scale ranging from 0 to 3. Score on the BDI-II is calculated from the sum of these items. Possible scores are between 0 and 63. Data from the BDI-II were gathered as the primary measure of depression symptom severity.

# Neuroimaging Data Extraction

The publicly available neuroimaging data from NKI-RS were extracted from an Amazon Web Services S3 bucket. High-resolution T1-weighted structural images were acquired for cortical thickness and brain volumetric analyses. Resting-state blood oxygen level-dependent functional images were extracted for functional brain network analyses. MRI acquisition and preprocessing procedures are detailed in subsequent sections. Quality control procedures were conducted on raw structural and functional MRI data based on guidelines provided by the University of Miami Brain Connectivity and Cognition Laboratory (<http://fcon_1000.projects.nitrc.org/indi/enhanced/qc.html>).

# Sample Determination

The workflow for determining the eligible participant pool is illustrated in Figure S1. Phenotypic data were extracted from LORIS for all participants between the ages of 18-85 years of age (n = 1,087). From this sample, participants with missing lab test data, diagnostic summary information, medical history, and/or medication data were excluded (n = 80). Diagnostic summary and medical history data were used to exclude participants who met any of the following criteria (n = 204): current or past alcohol or drug dependence; diagnosis of bipolar disorder, schizophrenia, or psychotic disorder; history of stroke, epilepsy, or convulsions/seizures; neurodegenerative disease (i.e., Alzheimer’s dementia, Parkinson’s disease, or Huntington’s disease); or diagnosis of autism, Asperger’s, or pervasive developmental disorder. Those who reported a duration of use < 3 months for currently prescribed psychotropic medications (e.g., stimulants, antidepressants) were also excluded (n = 13). Application of these exclusion criteria yielded a pool of n = 790 eligible participants.

Because planned analyses required complete data for each case entered into multiple linear regression models, participants from this initial sample were included only if PA measures, V̇O_2max_, BDI-II score, and both structural and functional neuroimaging data were available (n = 372). Participants were also excluded if > 28 days elapsed between collection of behavioral or neuroimaging data (n = 97). Quality control of the raw and preprocessed functional volumes resulted in exclusion of 49 additional participants prior to conducting group analyses. An additional nine participants were excluded based on casewise regression diagnostics. Details of these procedures are described in the subsequent sections. The final sample included n = 217 participants (134 females, 83 males) between the ages of 18 and 71 (*M* = 43.8, *SD* = 16.2; 76% white, 13% black or African-American, 9% Asian, 2% other race; 8% Hispanic; Figure S1).


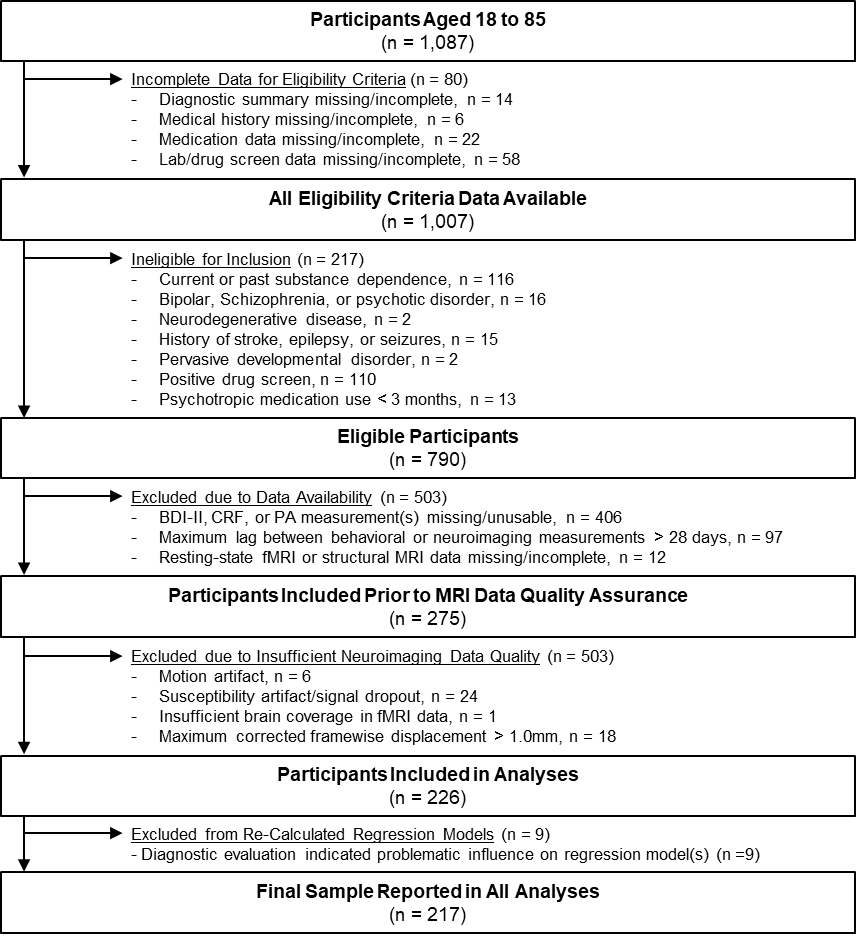


Figure S1. Workflow of inclusion and exclusion criteria for determination of eligible participants and inclusion of data in reported analyses

Functional MRI Data Quality Check. Raw T2*-weighted functional images were visually inspected for quality prior to conducting preprocessing procedures. Raw data from 31 participants were excluded from functional analyses due to insufficient brain coverage (n = 1) or moderate-to-severe motion (n = 6) or signal (n = 24) artifact. Common signal artifacts included signal dropout in the inferior temporal or frontal lobes, wrapping artifact, and ghosting that affected primarily anterior and posterior aspects of the raw images. Twelve participants had missing or unusable functional or structural MRI data and were therefore excluded.

Preprocessing Pipeline. Preprocessing of structural and functional MRI data was conducted using FreeSurfer and Analysis of Functional NeuroImages (AFNI). The transformation of each participant’s native space image to standard space (i.e., spatial normalization) was calculated using a multi-step process. Raw T1-weighted structural images were intensity-normalized within the FreeSurfer pipeline. Skull-intact and intensity-normalized T1-weighted images were crudely masked using a dilated mask derived from the FreeSurfer-estimated brain mask. This step ensured removal of extraneous data within the field of view (e.g., large sections of neck) that proved problematic for skull-stripping and warping. The nonlinear warp from each participant’s native space to standard Montreal Neurological Institute (MNI) space was then calculated using AFNI’s *3dQwarp* via *@SSwarper*. Briefly, skull-intact and intensity-normalized structural images were nonlinearly warped to a skull-on MNI template, cropped using a dilated brain mask from the MNI template, and combined with a first-pass skull-stripped version of the participant’s structural image to better estimate the native-space skull-stripped result. This skull-stripped image was then re-warped to the skull-stripped MNI template using concatenated 12-parameter affine and nonlinear transformations. For the current project, spatially normalized structural images were reviewed for quality control purposes, and the calculated affine and nonlinear warp matrices were saved for later transformation of subject-level network masks.

The first five functional volumes (i.e., TRs) collected in each run were discarded to account for scanner equilibration effects. The remaining volumes were despiked and slice-timing corrected to the beginning of the TR using Fourier interpolation. For motion correction, functional volumes were coregistered to the in-run volume with the minimum outlier fraction using a 6-parameter rigid-body affine transformation (AFNI’s *3dvolreg*) producing estimates of linear and rotational motion to be used in the regression model. Censoring, bandpassing, and nuisance regression were subsequently conducted simultaneously in one regression model. Within this model, data were bandpass filtered (0.01 to 0.1 Hz) and volumes with an outlier fraction > 0.05 were censored. Six estimated motion parameters and terms to account for variance due to scanner drift, signal from white matter and ventricles, demeaned motion, and motion derivative parameters were included in nuisance regressors. The resulting residual time series were scaled by a constant such that the mean in-run value at each voxel was 100. The affine transformation for alignment of native space and coregistered skull-stripped functional images with the high-resolution T1-weighted structural image was calculated in AFNI’s *align_epi_anat.py* using the lpc+ZZ cost function.

Quality Control of Preprocessed Functional MRI Data. Images were visually inspected after each step of the processing pipeline to ensure the quality of skull-stripping, alignment, normalization, etc. and corrected on a case-by case basis using modified scripts and/or adjustment methods. Based on motion estimates calculated by AFNI’s 3dvolreg, participants were excluded if their maximum framewise displacement (i.e., motion between successive TRs) was greater than 1.0 mm (n = 18). Residual motion after coregistration was minimal (average maximum framewise displacement ~ 0.0226 mm).

# Multiple Linear Regression Analysis

Pearson’s correlation coeffients for behavioral variables included in regression models are shown below.

Table S2. Correlation matrices for behavioral variables included in regression models.

| **FEMALES (n = 134)** |  |  |  |  |
| --- | --- | --- | --- | --- |
|  | **BDI-II** | **V̇O_2max_** | **PA** | **Age** |
| **BDI-II Score** | 1.00 |  |  |  |
| **Estimated V̇O_2max_** | -0.09 | 1.00 |  |  |
| **Total PA** | -0.10 | 0.12 | 1.00 |  |
| **Age** | 0.06 | -0.31 | 0.06 | 1.00 |
|  |  |  |  |  |
| **MALES (n = 83)** |  |  |  |  |
|  | **BDI-II** | **V̇O_2max_** | **PA** | **Age** |
| **BDI-II Score** | 1.00 |  |  |  |
| **Estimated V̇O_2max_** | -0.04 | 1.00 |  |  |
| **Total PA** | -0.11 | -0.13 | 1.00 |  |
| **Age** | -0.02 | -0.55 | 0.16 | 1.00 |
|  |  |  |  |  |
